# Domain Altering SNPs in the Human Proteome and Their Impact on Signaling Pathways

**DOI:** 10.1371/journal.pone.0012890

**Published:** 2010-09-23

**Authors:** Yichuan Liu, Aydin Tozeren

**Affiliations:** Center for Integrated Bioinformatics, Drexel University, Philadelphia, Pennsylvania, United States of America; Aarhus University, Denmark

## Abstract

Single nucleotide polymorphisms (SNPs) constitute an important mode of genetic variations observed in the human genome. A small fraction of SNPs, about four thousand out of the ten million, has been associated with genetic disorders and complex diseases. The present study focuses on SNPs that fall on protein domains, 3D structures that facilitate connectivity of proteins in cell signaling and metabolic pathways. We scanned the human proteome using the PROSITE web tool and identified proteins with SNP containing domains. We showed that SNPs that fall on protein domains are highly statistically enriched among SNPs linked to hereditary disorders and complex diseases. Proteins whose domains are dramatically altered by the presence of an SNP are even more likely to be present among proteins linked to hereditary disorders. Proteins with domain-altering SNPs comprise highly connected nodes in cellular pathways such as the focal adhesion, the axon guidance pathway and the autoimmune disease pathways. Statistical enrichment of domain/motif signatures in interacting protein pairs indicates extensive loss of connectivity of cell signaling pathways due to domain-altering SNPs, potentially leading to hereditary disorders.

## Introduction

Hereditary disorders are often linked to rare mutations in the form of single nucleotide polymorphisms (SNPs) [1]. Evolutionary forces introduce many new variants into the human genome in each generation [2]. SNPs affect the tendency to develop autism, diabetes, and cancer and impact immune response to pathogens, chemicals, drugs, and vaccines [3], [4], [5]. The HAPMAP project presents information concerning genetic variances among ethnic population subtypes thus implicating SNPs as key differences across population subtypes [6].

More than ten million SNPs have been identified in the human genome [7]. SNPs that fall into coding or promoter regions of proteins comprise only a small fraction of the presently annotated SNPs. To date, nearly four thousand SNPs have been mapped to the disease/disorder status [8]. Genome wide association studies complement clinical studies correlating SNPs to disease. However, approaches based on statistics alone provide limited insights on how a genetic variation causes disease.

Current methods for discovery of SNP-phenotype association include those focusing on non-synonymous SNPs that alter functional motifs such as binding sites [9], DNA binding motifs [10] and sites related to protein stability and cellular processing [11]. Computational intelligence models [12] utilize logic tree [13], [14], neural networks [15], an ensemble learning approach [15] or evolutionary algorithms [16] to discover correlations between SNPs and hereditary disorders and provide potential biological insight for the observed correlation and/or causation. Overall, the aforementioned approaches have illustrated the potential use of computational system modeling in the discovery of links between disease and the genotype.

This study focuses on a specific subset of human genotype-disease association, namely the annotation of SNPs that alter protein domains and thus potentially break bonds between interacting proteins in cell signaling pathways [17]. Protein domain structure is relatively flexible with respect to the amino acid sequence defining the domain, as illustrated by the domain annotation web tools such as Pfam [18] and PROSITE [19]. However, scanning proteins through these web tools, one can illustrate that even a single SNP could alter the structural configuration so extensively as to erase a domain from the structural composition of a given protein.

In this study, we screened the human proteome for domain annotation using the PROSITE web tool [20]. We projected the previously annotated SNPs onto proteins and identified those SNPs with domain altering properties. The resulting set turned out to be highly statistically enriched among proteins linked to genetic disorders [19]. We annotated these proteins using a variety of bioinformatics databases and web tools and showed that proteins with domain altering SNPs crowd the protein networks involving focal adhesion, axon guidance, natural killer cell mediated cytotoxity, and neurotrophin signaling pathways. Our predictions of linkages broken in these pathways indicate severe reduction of connectivity in signaling pathways associated with complex diseases and hereditary disorders.

## Methods

### Discovery of domain-altering SNPs

The human SNP database was downloaded from the NCBI dbSNP database (build 130, released June 2009) [7]. dbSNP is an archive containing over 10 million human SNPs, and among them, 63,899 missense SNPs. The SNPs were then projected onto corresponding human protein sequences from the NCBI GenBank. Peptide sequences of potentially SNP-containing proteins (in SNP-absent and SNP-containing forms) were screened for annotation of protein domains using PROSITE (version 20.31) [19]. A domain with potential to contain a SNP was called D-SNPs.

The PROSITE output for each sequence was in the form of a matrix with three columns, with the columns indicating (1) the ID number of the PROSITE domain, (2) the binary value identifying the presence or absence of a domain, and (3) the PROSITE matching score (MS) if the domain was expressed as a profile (position weight matrix) rather than expressed in the form of a pattern (regular expression). The second and the third columns of the output data allowed us to identify those SNPs that either removed a domain from the protein structure or drastically altered it when compared to sequences with and without the SNPs (DA-SNPs). If the PROSITE domain was defined as a regular expression, the second column was sufficient to identify whether the domain also existed in the presence of the SNP.

For those domains expressed as a profile, we checked the third column for the value of the matching score parameter of the same protein with and without the SNP. We defined a domain distortion (DD) parameter as the ratio of the difference in the matching score (due to the presence of the SNP) to the matching score of the sequence without the SNP. In our scans, DD varied from 0 to 0.3, which was the maximum domain distortion observed in our computations. Domains with DA-SNPs were defined as the sets of domains for which the sequence with SNPs no longer fits the regular expression, plus a set of profile domains with a finite DD value cut off in the presence of the SNPs.

Examples of structural diagrams of proteins with DA-SNPs were obtained using the Protein Data Bank (PDB, April 2010 version) [21] (for the case of no SNP) and SNPs3D [22] (with SNPs, 2008 version). The structures were aligned using YASARA [23] and the location of the SNP was marked with yellow. The lists of proteins with D-SNPs and DA-SNPs were presented as inputs to DAVID Bioinformatics resources [24] (version 6.7) and enriched KEGG pathway [25] profiles and Gene Ontology [26] categories at a p-value cut-off of 0.01.

### Bonds broken between a protein with a domain altering SNP and its neighbors in signaling pathways

We used statistical enrichment to identify protein signatures (domains, motifs) most likely to be found among binding partners of the proteins containing domain-altering SNPs. We created a score matrix with rows indicating domains that can be altered by an SNP and columns indicating domains and motifs found in binding partners of proteins with domain altering SNPs. Each element of the score matrix represented the number of times a domain with an SNP was found associated with a signature (domain, motif) on a binding partner. The web tool ELM (2010 version) [27] was used to annotate linear motifs on proteins. We then created random protein binding partners to proteins with SNP containing domains and created a score matrix as a background for statistical enrichment analysis. We used the hypergeometric test to identify those domains/motifs most likely to signal a protein-protein interaction involving domains with DA-SNPs. This procedure allowed us to identify signature pairs (such as A-B) such that the presence of signature A (domain with an SNP) in protein K and signature B in protein L would predict a binding interaction between K and L. The link A-B is a candidate for a bond potentially broken due to the presence of the domain altering SNP in a cellular pathway. To eliminate possible false positives in the estimates of bonds broken, we required the signature pair (A-B) to be either in the DOMINE (version 2.0) [28] database or previously annotated as a domain-motif pair as predictive of binding interactions between two proteins [29].

## Results

Our computations show that proteins with SNPs in one or more of its domains are significantly more likely to be associated with human disorders. Out of the 63,899 SNPs in the coding regions of proteins, 1,782 SNPs are present in the Online Mendelian Inheritance in Man (OMIM) database version 2009 [8]. A total of 12,965 SNPs fall into protein domains, and 592 proteins with domain SNPs are associated with a disease or disorder in OMIM. The p value from hypergeometric test for the SNP enrichment within the domain regions is zero, which indicating that SNPs in the domain regions of proteins are highly correlated to genetic disorders and complex diseases. This observation is consistent with the important functions protein domains play in establishing connectivity among proteins in cell signaling pathways [17].

**Figure 1:**
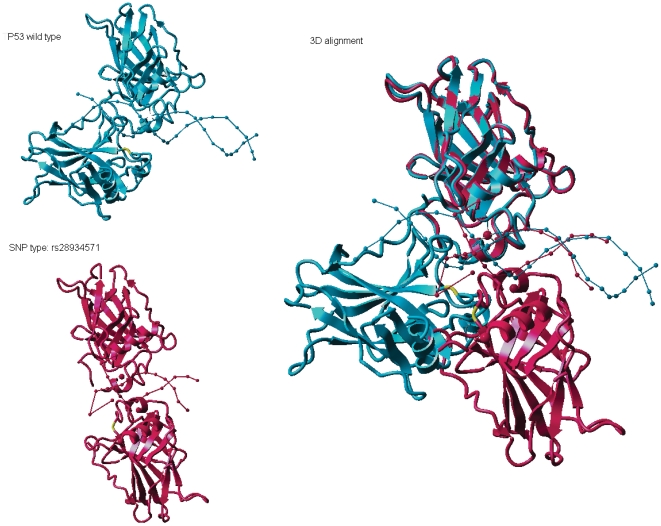
Alteration of 3D structure of TP53 due to presence of SNP rs28934571. The TP53 protein wild type (light blue) and the same protein with the SNP (pink) are shown on the left, and their optimal alignment on the right. The SNP position *is* colored as yellow in resulting structure.

Among proteins with domain SNPs, those with domain-altering SNPs are even more likely to be associated with disorder/disease. Domain-altering SNPs discovered in our PROSITE screening method consists of two subsets. The first subset consists of SNPs the sequence no longer satisfies in the regular expression for the domain. The second subset is composed of domains defined as a profile above a prescribed domain distortion (DD) parameter cutoff. An example of a domain with DA-SNP is p53. Figure 1 shows the 3D structure of TP53 in the presence and absence of a DA-SNP (SNP rs28934571), as well as the poor alignment of these structures due to the presence of the SNP. This SNP occurs at sequence position 249 and causes losses of hydrogen bonds and salt bridge bonds. The root mean square deviation (RMSD) [30] between TP53 and its DA-SNP structure is 52.6496 A. Another example of a domain-altering SNP is SNP rs29001653. This SNP alters the visual pigments retinal binding site of the protein coded by the RHO gene, resulting in night blindness. The altered structure is over packed in the 3D space and the RMSD is 2.9835 A. Because 3D domain structure is not available for most domains with synonymous SNPs at present time, it is not possible to define domain-altering SNP based on the RMSD evaluations, and hence the use of domain-altering SNP definition described in the methods section.

We evaluated the statistical enrichment of the domain altering SNPs in the OMIM Database as a function of the DD cutoff, as shown in Table 1. In all cases, the p value <0.05 indicates significance of enrichment with respect to all domains with SNPs. The list of proteins with domain-altering SNPs in which both the protein and the SNP were linked to the same disease/disorder is presented in Table S1 for DD >0.10. The phenotypes were identified by reading through the OMIM disease/disorder documentations. The table covers proteins associated with a variety of diseases ranging from pancreatic cancer, epilepsy, and to carpal tunnel syndrome. Beside the OMIM evaluations, SNPs reported in various cancer lines were tested [31] for their position relative to domains and domain altering potential. Out of 13 unique SNP IDs presented in Table 2 of Zhang [31], one was found in the PROSITE domain region, and it corresponded to a domain-altering SNP associated with pancreas cancer.

**Table 1:** Statistical enrichment of domain altering SNPs in the OMIM database.

# D-SNP	# D-SNP & OMIM	DD cut-off	# SNPs	OMIM match	p value
12965	801	0.05	1152	75	0.0444
		0.1	598	46	0.0197
		0.15	497	40	0.016
		0.2	451	35	0.028

Each row gives the overall statistics of domain-altering SNPs and the p value for statistical enrichment in OMIM at a given domain distortion (DD) parameter cutoff. The p values are computed based on hypergeometric test.

**Table 2:** The top ten most highly connected proteins with domain altering SNPs (DD>0.10).

Gene	Protein Name	# broken	# intact
ADRBK1	beta-adrenergic receptor kinase 1	29	9
SH2D1A	SH2 domain-containing protein 1A	12	0
TCF3	transcription factor E2-alpha	19	25
APBA2	amyloid beta A4 precursor protein-binding family A	8	1
NCK1	cytoplasmic protein NCK1	61	3
PRKCH	protein kinase C	8	0
YWHAE	14-3-3 protein epsilon	46	21
TOPBP1	topoisomerase (DNA) II binding protein 1	11	3
BCAR1	breast cancer anti-estrogen resistance protein 1	45	3
RIMS1	regulating synaptic membrane exocytosis protein 1	11	3

**Figure 2:**
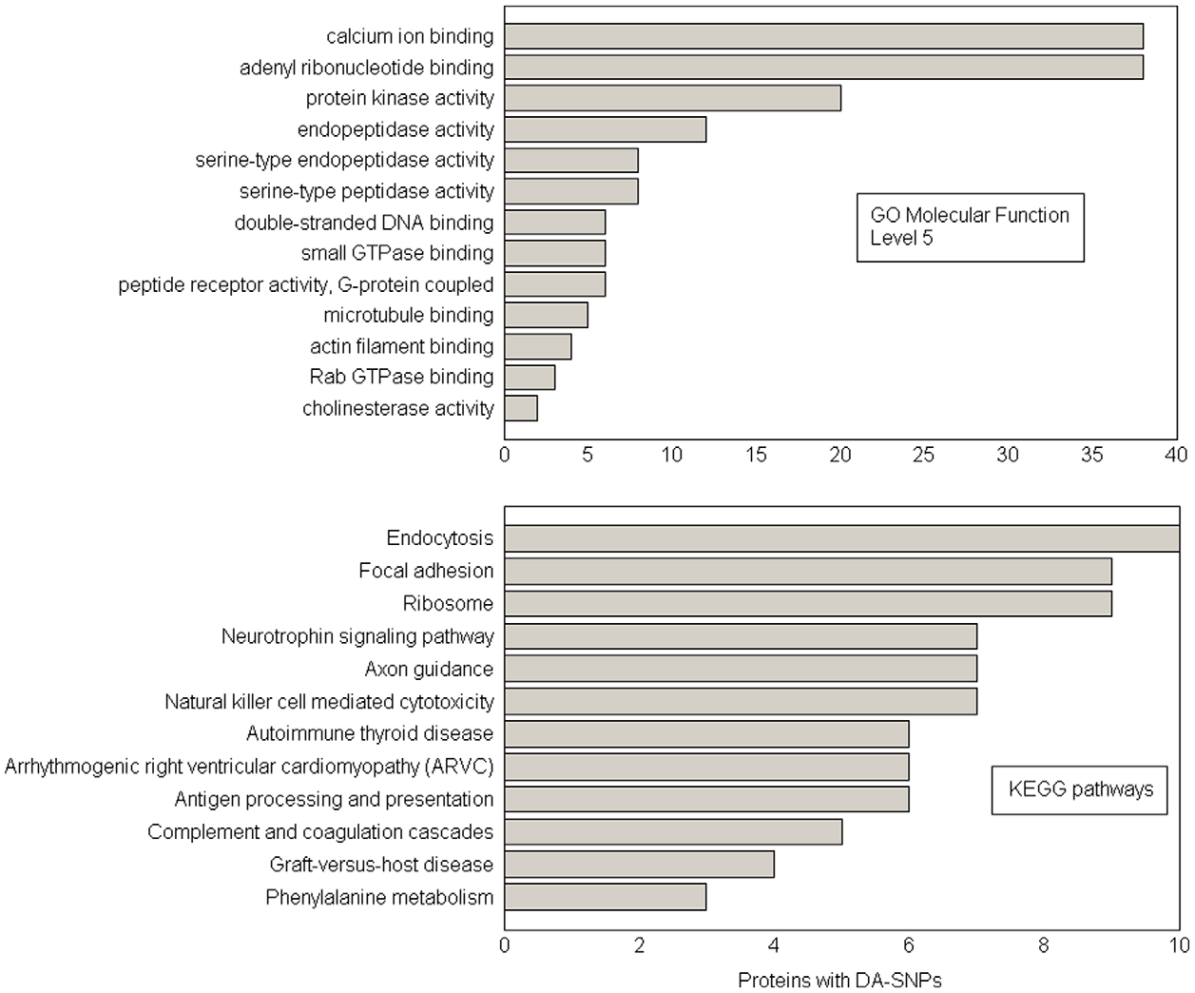
Statistically enriched Gene Ontology (GO) molecular function level 5 (MF) categories (2A) and KEGG cellular pathways (2B) for DD >0.10 at p value <0.01.

Proteins with domain-altering SNPs crowd GO molecular function categories involving calcium ion binding, adenyl ribonucleotide binding, protein kinase activity, and endopeptidase activity at DD >0.10. The DD cutoff of 0.10 corresponds to 598 domain-altering SNPs present in 505 proteins. Among these proteins, 242 had at least one known binding partner in the Human Protein Reference Database (HPRD) [32]. The GO level 5 molecular function gene ontology categories shown in Figure 2A are statistically enriched with proteins with domain altering SNPs (p<0.01). The list of GO categories shown in the figure indicates that proteins with domain-altering SNPs comprise key nodes in protein networks; their loss of connectivity would likely have a significant effect on cellular signal transduction.

**Figure 3:**
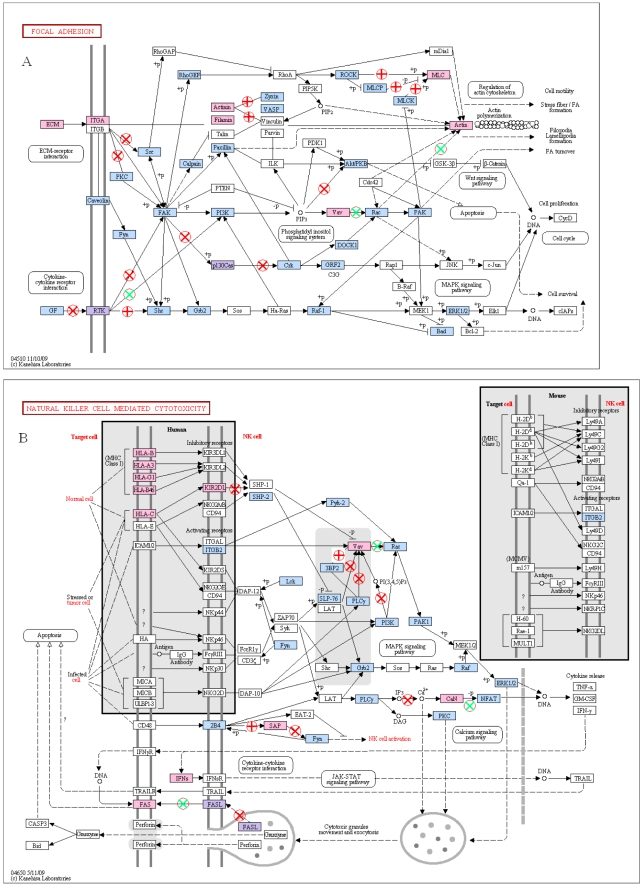
Proteins with domain altering SNPs on KEGG pathways for focal adhesion (3A) and natural killer cell mediated cytotoxicity (3B). Also shown these diagrams are the broken edges (links) estimated by statistical enrichment of domain pairs in protein-protein interactions (circles containing + or x signs.

Shown in Figure 2B is the list of KEGG pathways statistically enriched (p<0.01) in proteins with domain-altering SNPs at DD >0.10. The list contains pathways closely associated with cancer, neurological, and immunological diseases. Proteins with domain altering SNPs are marked in the pathways for focal adhesion and natural killer cell mediated cytotoxicity in Figure 3. Nodes colored in pink in these figures indicate proteins with domain-altering SNPs, while those in blue are their immediate binding partners as identified in HPRD. The purple nodes are proteins that belong to both the pink and blue groups. The pathway diagrams shown in Figure 3 illustrate the presence of proteins with domain altering SNPs from the very beginning of the pathway at the cell membrane all the way to the transcription factors regulating important cellular processes.

Next, we estimated the links broken in these pathways due to domain-altering SNPs. We have determined domain-domain and domain-motif pairs (signature pairs) statistically enhanced in protein-protein interactions involving proteins with domain-altering SNPs. The results show that only a very small fraction of possible signature pairs are statistically enriched in PPIs presented in HPRD [26].

**Figure 4:**
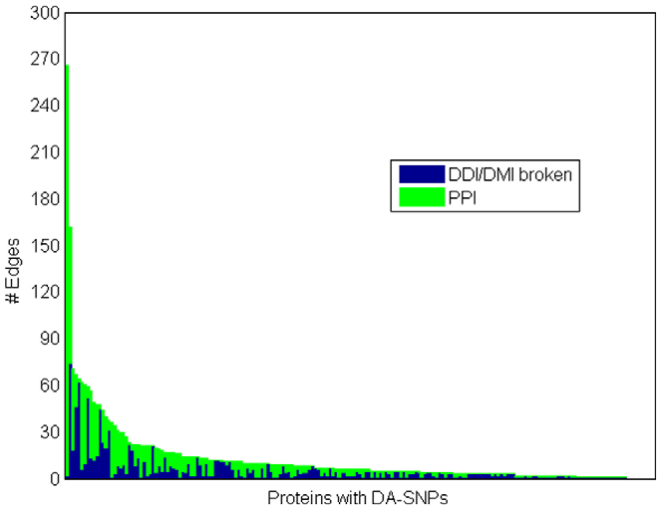
Figure 4 shows a histogram for proteins containing a domain-altering SNP indicating the number of edges each has in the absence of SNP, and the estimate of broken edges in the presence of the SNP.

We used statistically enriched signature pairs in the estimates of links broken due to a protein expressing a domain-altering SNP. A link (transient or stable) between two proteins is assumed broken due to a domain altering SNP if the opposing protein pair contains at least one signature enriched with the SNP containing domain in the PPIs in HPRD. Shown in Figure 4 is the histogram for proteins with DA-SNPs, thus indicating the number of edges such proteins have in the absence of an SNP and the number of edges estimated to be broken due to the presence of the SNP. The figure indicates potentially extensive loss of connectivity of proteins with DA-SNPs to neighboring proteins in protein networks.

The protein links we estimate to be broken between a protein containing a DA-SNP and its immediate neighbors are shown in Figure 3A and 3B for focal adhesion and natural killer cell mediated immunity. The links deemed to be broken using the statistical enrichment method described above are shown as marked with a circle containing either a red or green “x” or a red “+” sign. The links with red “x” correspond to the domain signature pairs present in the DOMINE database described as predicting PPIs with high accuracy [27]. The links with a green “x” correspond to domain-motif associations deemed highly predictive of PPIs [25]. The links marked (+) are links estimated to be broken by statistical enrichment of the domain pairs only. Note that, even when we exclude these latter links, the pathway connectivity (number of edges per node) is strongly influenced when an SNP drastically alters the 3D shape of a domain responsible for connections to upstream and downstream partners. This assertion is further enhanced by the list of highly connected proteins with domain altering SNPs given in Table 2. For example, the transcription factor TCF3 has 44 known binding partners, and links to 19 of these partners could be broken due to the presence of the domain-altering SNP. Taken together, our study exposes the importance of domain SNPs in the progression of some of the most prominent complex and/or hereditary diseases.

## Discussion

The genome wide association studies seeking a correlation between genetic makeup and complex diseases, such as cardiovascular diseases [33], autism [34], and diabetes [35]. Results implicate a handful of SNPs correlated with these complex disease states. Correlation is based on purely statistical methods, and in many cases, SNPs found to be significantly associated with a disease fell into the non-coding regions of DNA distant from a protein coding gene [12]. As the population subsets for genome-wide studies grow in size with increasing research efforts and time, and as these sets are better controlled for demographic and environmental variables, one would expect the discovery of sets of additional SNPs strongly correlated with hereditary disorders and disease subtypes. Nevertheless, a system bioinformatics approach is needed to explore how a disease-correlated SNP alters cell signaling and metabolic pathways, thus contributing to the initiation of a disorder or a disease.

This study explores the mechanisms by which SNPs that fall into protein domains in the human genome potentially contribute to disease. Protein domains are functional units closely aligned with post-transcriptional modification (as in phosphorylation) and play important roles in establishing the connectivity of cell signaling networks via binding to upstream and downstream proteins [4], [36]. Our computations indicate that the p value for disease association of SNPs that fall into protein domains and occur by random chance is practically zero. Within this group of SNPs, those with domain-altering properties are even more likely to be associated with a disease state. We have defined a domain-altering SNP as one that either alters the sequence such that it no longer satisfies the regular expression of the domain or that the domain is extensively deformed as quantified by the domain distortion index. Proteins with domain-altering SNPs crowd cellular pathways involved in neurological, and immunological diseases, as well as in cancers such as the pancreatic cancer.

How does an SNP with domain altering properties affect the connectivity of pathways? The key to answering this question lies in the discovery of the set of proteins that bind to proteins under consideration via the domain containing the SNP. The grammar of protein-protein interactions in terms of primary sequence and/or 3D structure is yet to be fully understood. We used a statistical enrichment approach to identify protein domains (motifs) on the opposing protein most frequently associated with the SNP-containing domain under consideration. We then assumed a bond (transient or steady) was broken whenever we came across such a signature pair among the immediate partners of the protein with a domain altering SNP. Results shown in the present study for focal adhesion and the natural killer cell mediated cytotoxicity pathways indicate extensive loss of connectivity in these cellular pathways, caused by the presence of domain altering SNPs among the proteins in these pathways. Even when we reduced the estimates of bonds broken with the use of signature pairs already known to predict protein-protein interactions, the loss of connectivity persisted at multiple cell compartments. We obtained qualitatively similar results for axon guidance and neutrophin signaling pathways altered by the presence of a domain altering SNP (not shown).

In conclusion, proteins with domain-altering SNPs are statistically enriched in the list of proteins known to be associated with disease. These proteins crowd pathways associated with immunological, neurological and cardiomyopathy disorders. Protein functional groups statistically enriched with proteins with domain altering SNPs include calcium ion binding, adenyl ribonucleotide binding, protein kinase activity, endopeptidase activity, serine-type peptidase activity, DNA binding, and GTPase binding proteins.

## Supporting Information

Table S1 List of proteins with domain altering SNPs (DD >0.1) for which both the SNP and the protein were previously associated in the literature with a hereditary disorder or complex disease. The columns in the table present the gene symbol, the SNP ID, the name of the domain with the SNP, whether is DD violation (yes) or violation of regular expression (no), and the associated disorder/disease.
(0.02 MB XLS)
